# Cost-effectiveness of empagliflozin in patients with type 2 diabetes and established cardiovascular disease in China

**DOI:** 10.1186/s12962-021-00299-z

**Published:** 2021-08-04

**Authors:** Mafalda Ramos, Peng Men, Xu Wang, Anastasia Ustyugova, Mark Lamotte

**Affiliations:** 1 IQVIA Global HEOR, Lagoas Park, Edifício 3 - Piso 3, 2740-266 Porto Salvo, Portugal; 2 Department of Pharmacy, Peking University Third Hospital, Beijing, 100191 China; 3 Institute for Drug Evaluation, Peking University Health Science Center, Beijing, 100191 China; 4 Boehringer Ingelheim, Beijing, China; 5 Boehringer Ingelheim, Ingelheim Am Rhein, Germany; 6 IQVIA, Global HEOR, Zaventem, Belgium

**Keywords:** Cost-effectiveness, Empagliflozin, Type 2 diabetes, Cardiovascular outcomes, Core Diabetes Model

## Abstract

**Background:**

In several cardiovascular outcome trials (CVOTs), empagliflozin (SGLT-2 inhibitor), sitagliptin (DPP-4 inhibitor) and liraglutide (GLP-1 receptor agonist) + standard of care (SoC) were compared to SoC in patients with type 2 diabetes and established cardiovascular disease (CVD). This study assessed the cost-effectiveness (CE) of empagliflozin + SoC in comparison to sitagliptin + SoC and liraglutide + SoC based on the respective CVOT.

**Methods:**

The IQVIA Core Diabetes Model (CDM) was calibrated to reproduce the CVOT outcomes. EMPA-REG OUTCOME baseline characteristics and CVOT specific treatment effects on risk factors for cardiovascular disease (HbA1c, BMI, blood pressure, lipids) were applied. Three-year observed cardiovascular events of empagliflozin + SoC versus sitagliptin + SoC and liraglutide + SoC were derived from EMPA-REG OUTCOME and an indirect treatment comparison. Relative risk adjustments to calibrate the CDM were obtained after a trial and error process to match as closely the observed and CDM-predicted outcomes. The drug-specific treatment effects were considered up until HbA1c reached 8.5% and treatment switch occurred. After this switch, the United Kingdom Prospective Diabetes Study 82 risk equations predicted events based on co-existing risk factors and treatment intensification to basal bolus insulin were applied. The analysis was conducted from the perspective of the Chinese healthcare system applying 3% discounting. The time horizon was lifelong.

**Results:**

Empagliflozin + SoC provides additional Quality Adjusted Life years (QALY + 0.564) for an incremental cost of 42,497RMB (US$6053) compared to sitagliptin + SoC, resulting in an Incremental Cost Utility Ratio of 75,349RMB (US$10,732), thus below the willingness-to-pay threshold of 212,676RMB, corresponding to three times the Gross Domestic Product in China (2019). Compared to liraglutide + SoC, empagliflozin + SoC use leads to 0.211QALY gained and cost savings of 71,427RMB (US$10,173) and is as such dominant. Scenario and probabilistic sensitivity analyses demonstrated the robustness of the results.

**Conclusion:**

Results suggest that empagliflozin + SoC is cost-effective compared to sitagliptin + SoC and liraglutide + SoC at a willingness-to-pay threshold of 212,676RMB ($30,292)/QALY.

**Supplementary Information:**

The online version contains supplementary material available at 10.1186/s12962-021-00299-z.

## Background

Type 2 diabetes (T2D) is a heterogeneous syndrome caused by the interaction of environmental factors with multiple diabetogenic genes, which lead to various combinations of insulin resistance and islet β-cell failure [1]. According to the International Diabetes Federation, around 463 million adults were living with diabetes globally in 2019, and T2D accounted for almost 90% of all the diabetes cases [2]. China bears the highest prevalence of diabetes (116.4 million) in the world, of which 10.4% are adults [3]. The prevalence of diabetes has grown alarmingly in China from 5.5% in 2001 [4] to 10.9% in 2013 [5]. The increased clinical burden of T2D has also translated into considerable health expenditure on T2D in China, increasing from USD 0.25 billion in 1993 [or 0.07% of Gross Domestic Product (GDP)] to USD 8.65 billion in 2008 (or 0.21% of GDP) [6].

It is noteworthy that the health expenditure of patients with diabetic complications was 3.36 times higher than those without complications in China [7]. Evidence suggests that T2D is often linked with increased cardiovascular (CV) risk [8, 9] and CV disease (CVD) is considered the major comorbidity leading to premature death in patients with T2D [10]. This well-known correlation between T2D and CVD compelled the US Food and Drug Administration (FDA) in 2008 [11] and the European Medicines Agency (EMA) in 2012 [12] to demand the conduct of post-marketing CV outcome trials (CVOTs) that focus on controlling the risk of CVD, besides targeting a decrease of glucose and glycated hemoglobin (HbA1c) levels in patients with T2D [13]. In addition, the 2019 treatment guidelines for T2D provided by the Chinese Diabetes Society identifies hypertension (systolic blood pressure ≥ 140 mmHg and/or diastolic blood pressure ≥ 90 mmHg) or use of antihypertensive therapy as one of the definitions of high risk populations with diabetes [14].

Over the past two decades, several CVOTs have demonstrated reductions in the risk of CV outcomes and mortality in patients with T2D. A review of CVOTs completed between 2008 and 2016 demonstrated that new glucose-lowering drugs such as dipeptidyl peptidase 4 (DPP4) inhibitors (saxagliptin, alogliptin and sitagliptin), glucagon-like peptide 1 (GLP-1) receptor agonists (lixisenatide, liraglutide, and semaglutide) and sodium-glucose co-transporter 2 (SGLT-2) inhibitor (empagliflozin) do not increase the CV risk in patients with T2D compared to standard of care (SoC) [13]. Not only were these CVOTs capable of proving CV safety, several trials, and in particular—EMPA-REG OUTCOME (empagliflozin + SoC) [15] and LEADER (liraglutide + SoC) [16] were able to demonstrate CV benefits even when they were primarily designed for non-inferiority. On the contrary, safety warnings have been issued by the US FDA on the increased risk of heart failure with saxagliptin and alogliptin, while no such concerns were observed with sitagliptin + SoC in the TECOS trial [17].

Empagliflozin is a selective SGLT-2 inhibitor that has been approved for HbA1c management in patients with T2D and has been listed in the National Reimbursement Drug List in late 2019, three years after approval by the Chinese National Medicinal Products Administration (i.e. Chinese FDA). However, due to the relatively higher prices compared to other conventional oral antidiabetic drugs, treatment with empagliflozin may impose a potential economic impact on the healthcare payer [18]. Despite the rapid increase in the prevalence and economic burden of diabetes in China, Mao and colleagues have highlighted that a national strategy or special financing mechanism to meet these challenges is lacking in China [19]. In addition, there is a lack of published evidence on pharmacoeconomic analysis of empagliflozin versus other antidiabetic drugs in China. Thus, our study was conducted with the aim of assessing the long-term cost-effectiveness of empagliflozin + SoC in adult patients with established CVD compared to liraglutide + SoC and sitagliptin + SoC in Chinese patients with T2D.

## Methods

### Model framework

#### IQVIA Core Diabetes Model

The IQVIA Core Diabetes Model (CDM) version 9.0 model was used to assess the long-term CE of empagliflozin + SoC in adult patients with established CVD in China. The CDM is a non-product-specific diabetes policy analysis tool that performs real-time simulations considering oral anti-diabetics (OADs), screening and treatment strategies for micro-vascular complications. The basic structure of CDM was described by Palmer et al. in 2004 [20] and the most recent validation of the model was published by McEwan et al. in 2014 [21]. Updated information and description of the model structure are available online (http://www.core-diabetes.com/).

The outcomes for the current analysis included life-years (LYs), quality-adjusted LYs (QALYs), cumulative incidence of diabetes-related complications, progression of physiologic parameters, and direct medical costs. The model was calibrated to reflect the clinical efficacy outcomes observed in the EMPA-REG OUTCOME trial for empagliflozin + SoC [15] and, due to the lack of head-to-head trial comparisons, from indirect treatment comparisons (ITCs) of empagliflozin + SoC to its comparators sitagliptin + SoC [22] and liraglutide + SoC [23]. The steps involved in this calibration process have been described in similar previous cost-effectiveness analyses conducted using CDM for the comparison of empagliflozin + SoC with liraglutide + SoC [16] and with sitagliptin + SoC and saxagliptin + SoC [17].

Three-year event rates for empagliflozin + SoC were taken directly from the EMPA-REG OUTCOME trial [15]. Event rates for sitagliptin + SoC and liraglutide + SoC were calculated using relative risks from their respective ITCs with empagliflozin + SoC [22, 23] (Table 1). In order to match the endpoints specified in the EMPA-REG OUTCOME trial and those reported by the ITCs with the CDM endpoints, some assumptions were made, which are summarized in Additional files 1 and 2.

**Table 1:** Result comparison of expected vs. projected 3-year cumulative incidence (%) outcomes for empagliflozin + SoC and comparators post CDM CV outcome calibration

Event	Empagliflozin + SoC		Sitagliptin + SoC		Liraglutide + SoC	
	From EMPA-REG OUTCOME	Calibrated	Estimated by ITC	Calibrated	Estimated by ITC	Calibrated
Death from any cause	5.82	5.78	8.69	8.67	7.28	7.24
Death from CV causes	3.72	3.68	6.20	6.13	4.65	4.63
MI	5.04	5.05	5.04	5.00	5.09	5.08
Angina	3.00	3.01	3.00	3.01	3.00	3.06
Stroke	3.69	3.70	3.69	3.69	2.72	2.71
HF	2.82	2.83	4.34	4.36	3.76	3.79
MAU	75.75	75.86	79.8	79.1	75.75	76.76
GRP	12.54	12.34	19.47	14.89	12.54	9.17
ESRD	0.3	0.3	0.63	0.54	0.3	0.28

*CDM *IQVIA Core Diabetes Model, *CV* cardiovascular, *ESRD* end-stage renal disease, *GRP* gross proteinuria, *HF* heart failure, *ITC* indirect treatment comparison, *MAU* microalbuminuria, *MI* myocardial infarction, *SoC* standard of care

### Clinical data

#### Patient characteristics

The cohort under analysis represents the characteristics of patient with T2D and established CVD at baseline, and as such, the baseline characteristics of patients in the EMPA-REG OUTCOME trial were used (Additional file 3) [20].

The CDM was calibrated to reflect the CV outcomes in EMPA-REG OUTCOME trial till treatment switch. The baseline data included cohort characteristics such as mean age, duration of diabetes and presence of co-morbidities like myocardial infarction (MI), stroke, heart failure (HF), microalbuminuria (MAU), proteinuria, end-stage renal disease (ESRD), retinopathy complications, foot ulcer and neuropathy.

#### Treatment effects

The impact of the treatments on the different risk factors were populated in the CDM as annual changes and aligned with each trial. First year benefit was programmed as change from baseline in the treatment settings, together with the associated adverse event rates of each treatment (Table 2).

**Table. 2:** First year treatment effects of first- and second line treatments

Variable	Empagliflozin + SoC [24, 25]	Sitagliptin + SoC [26]***	Liraglutide + SoC [27]	Basal Bolus [28]
HbA1c*	− 0.58	− 0.328	− 1.37	− 0.828
SBP*	− 3.9	− 0.62	− 1.82	0
DBP*	− 1.72	− 0.78	0.17	0
T-Chol*	7.81	3.56	0	0
HDL*	1.81	− 0.09	0	0
LDL*	4.79	1.42	0	0
TRIG*	0	0	0	0
BMI*	− 0.64	− 0.04	− 0.88	0.32
eGFR*	− 0.16	0.18	0	0
NSHE rate**	13.62	13.98	289.12	2566.83
SHE1 rate**	0.44	0.64	8.24	23.81
SHE2 rate**	0.06	0.14	1.10	3.19
GUI**	10.53	8.95	8.95***	–

*BMI* body mass index, *DBP* diastolic blood pressure, *eGRF* estimated glomerular filtration rate, *GUI* genital and urinary tract infection, *HbA1c* glycated hemoglobin, *HDL* high-density lipoprotein cholesterol, *HR* heart rate, *LDL* low-density lipoprotein cholesterol, *NSHE* non-severe hypoglycemic event, *SBP* systolic blood pressure, *SHE* severe hypoglycemic event, *SoC* standard of care, *T-Chol* total cholesterol, *TRIG* triglycerides

* Effect on the surrogate endpoints is applied on the first year of treatment

** Rate per 100 patient-year

*** Aside HbA1c effect, all other endpoints are assumed to be equal to placebo described in the EMPA-REG OUTCOME trial

For the 2nd and 3rd years, progression tables of risk factors were populated with post 1st year annual treatment effects up to the end of the respective study periods. It should be noted that the treatment effects described in each trial reflected the impact of both main therapy and the intensification of SoC, as specified in the respective study protocols. From the 4th year onwards, HbA1c progression reported with empagliflozin + SoC in the EMPA-REG OUTCOME study was applied to all competitors assuming that all therapies were equally effective. This choice was again to avoid the initial high impact on HbA1c levels reported in the LEADER study [16] and the slow increase of HbA1c over time, the latter primarily driven by the fact that most patients were receiving insulin.  

#### Treatment duration/switch to rescue therapy

The EMPA-REG OUTCOME and comparator CVOTs were designed to assess the effect of investigational drug versus placebo as an add-on to what can be considered SoC background therapy. This background therapy is a combination of other glucose-lowering drugs that were already administered at study initiation or escalated over the study duration. The background therapy was, therefore, diverse and evolving over time in the CVOT. Nevertheless, as the time horizon for this analysis was lifetime (50 years), it would be inappropriate and unrealistic to apply the reported combined therapies throughout the time horizon. As such, treatment switch was programmed when an 8.5% HbA1c threshold was reached, except for liraglutide + SoC. Patients in the liraglutide + SoC arm were forced to switch after eight years of treatment, the same as for empagliflozin + SoC, before they reach an HbA1c of 8.5% since the patients in the LEADER trial reported a large initial HbA1c decrease, followed by a slow decrease in HbA1c over time. As such, an HbA1c of 8.5% would only be expected after 13 years and switch of treatment in the liraglutide + SoC arm would also only be after 13 years. Hence, a treatment switch after 8 years was considered, as it aligned with the number of years needed for empagliflozin + SoC to reach an HbA1c of 8.5%; however, a switch after 13 years was considered in a scenario analysis.

All patients would switch to rescue therapy, which was defined as a basal bolus insulin therapy at a basal insulin (described as glargine 100 unit/mL) dose of equal to 59 units per day and bolus insulin equal to 36.9 units per day as reported by Riddle et al. [28]. Basal-bolus insulin therapy was considered to be the most plausible treatment escalation regimen since background insulin regimen was already provided to half of patients at Week 164 of the EMPA-REG OUTCOME trial (50% in empagliflozin + SoC and 58% in placebo). Given these characteristics, it was assumed that glargine as add-on to rapid acting insulin would be a good representation of the basal bolus therapy, as rescue therapy and best indicated as second line therapy for patients failing several combined treatments as add-on to insulin.

**Fig. 1:**
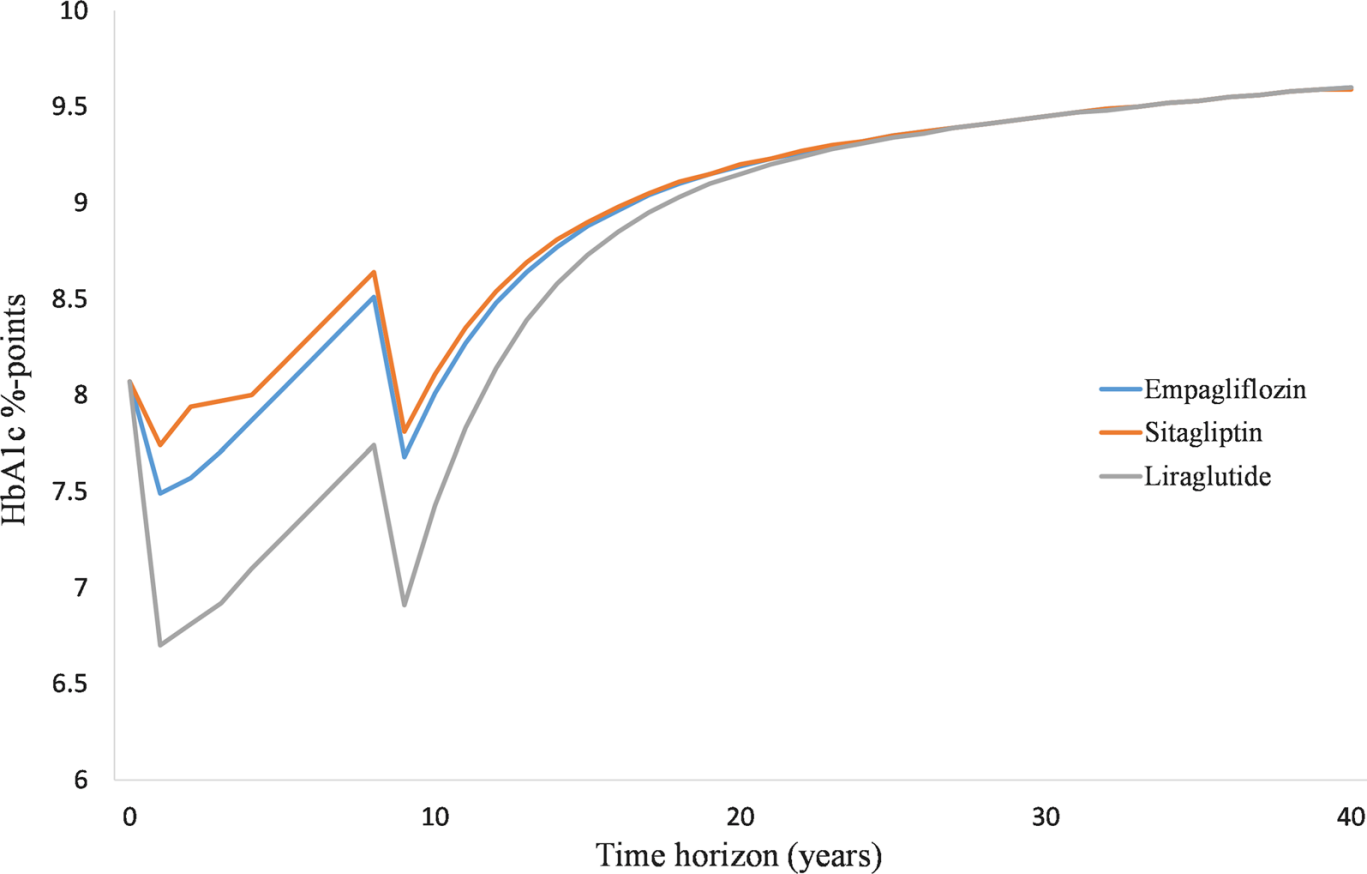
Progression of HbA1c over time. *HbA1c* glycated hemoglobin

HbA1c progression was programmed as per description in the different CVOTs and their follow-up periods. It was then linearly extrapolated (see the evolution of HbA1c per treatment arm in Fig. 1). Before treatment switch, mortality, CV and renal outcomes in the CDM are predicted as described in the calibration process [16, 17]. Thus, they matched the event rates as reported in the EMPA-REG OUTCOME trial and those determined with relative treatment efficacy derived from indirect comparison. After treatment escalation, the standard United Kingdom Prospective Diabetes Study (UKPDS) 82 Risk Equations [29] were applied to predict mortality and CV outcomes. The default CDM probabilities for developing renal disease were also applied.

### Cost data

This analysis was conducted from the Chinese healthcare payer’s perspective. As such, only direct costs were considered, and indirect costs were not included in the analysis. Direct medical costs included pharmacy costs, management cost (screening test, concomitant medication) [30] and the costs of T2D complications (CVD complications, renal complications, acute events, eye disease, neuropathy, foot ulcer and amputation) [30] (Additional file 4). Drug unit costs were extracted from the Yaozh website (Available from: https://www.yaozh.com/, 2020). All costs are inflated to 2019. The annual treatment cost per arm comprised of the main therapy cost as well as the cost of background insulin as concomitant therapy. The background treatment costs for insulin were included as insulin was the main adjuvant treatment commonly present in all included trials and is associated with a considerable cost (unlike metformin or sulfonylurea).

The first and subsequent years’ treatments cost of each alternative therapy under comparison plus the cost of treatment intensification on basal bolus insulin regimen are presented in Table 3.

**Table. 3:** First year and follow-up year treatment costs of each alternative (CDM inputs of treatment cost group setting; RMB)

Treatment	1st year	Subsequent years
Empagliflozin + SoC	9,682.79	10,057.76
Liraglutide + SoC	19,943.75	24,347.57
Sitagliptin + SoC	6,288.94	7,772.18
Basal bolus	19,866.77	19,866.77

*CDM *IQVIA Core Diabetes Model, *SoC* standard of care

### Utility data

Starting from a Chinese specific baseline utility [31], the default CDM utility data associated with events and health states derived from Beaudet et al. [32] were applied (Additional file 5). A minimum approach is applied to estimation of utilities, wherein the event with lower utility is applied whenever multiple events are accounted within an observation period (e.g. using utility value for stroke event if a patient has a history of both MI and stroke). In addition, the impact of change in BMI on utility was estimated through the inclusion of a disutility of -0.0061 per unit gain in BMI for over 25 kg/m^2^, based on utility values provided by Bagust and Beale [33]. It was assumed that BMI remained constant over time in all trial arms after treatment switch.

### Mortality data

For calculation of mortality, the UKPDS 82 mortality equations were applied. This approach was chosen as part of the calibration process and was leading to model predicted outcomes that came closest to the trial outcomes.

### Analysis plan

#### Base case analysis

In base case analysis, lifelong analysis over a time horizon of 50 years was taken into consideration. An annual discount rate of 3.0% was applied for both costs and effects, as recommended by the Chinese Center for Health Economics Research [34].

#### Uncertainty assessment

Scenario analysis and probabilistic sensitivity analysis (PSA) were conducted to assess the impact of changing key input variables on the CEM.

#### Scenario analyses

Scenario analysis involved a series of simulations, in which key input assumptions were tested to assess their impact on clinical and cost outcomes. Five different scenarios were assessed on perspectives related to exclusion of insulin cost from treatment costs, limiting CV outcomes to only three years, adjusting the time horizon to five years with CV outcomes applied through the five years, and setting an HbA1c threshold of 9%-points (at which patients switch to basal bolus insulin).

Additional analyses were also run for empagliflozin + SoC versus liraglutide + SoC, where the effects of liraglutide + SoC were not limited to eight years, but were applied until HbA1c of 8.5% is reached, at 13 years.

#### Probabilistic sensitivity analysis

The PSA was performed using the base case settings and a non-parametric bootstrapping approach in which the progression of diabetes was simulated in 1000 patients, each run through the model 1000 times so as to calculate the mean and standard deviation of costs, life expectancy and quality-adjusted life expectancy. Results are presented in the cost-effectiveness plane, and as cost-effectiveness acceptability curves (CEACs).

## Results

### Base case

Table 4 shows that empagliflozin + SoC provides additional LY and QALYs compared to both sitagliptin + SoC and liraglutide + SoC. In spite of incurring an incremental total cost of 42,497 RMB (US$6053) compared to sitagliptin + SoC, the use of empagliflozin + SoC results in incremental cost-utility ratios (ICURs) below the threshold of 212,676 RMB [three times the Chinese Gross Domestic Product per capita, as defined by World Health Organization (WHO)], and hence, empagliflozin + SoC can be considered cost-effective. Compared to liraglutide + SoC, empagliflozin + SoC use results in a total cost saving of 71,427 RMB (US$10,173) and as such, can be considered the dominant treatment alternative.

**Table. 4:** Cost-effectiveness results when CV outcomes are extended until treatment switch (per average patient)

Parameters	Empagliflozin + SoC	Sitagliptin + SoC	Liraglutide + SoC
LY (years)	10.673	9.901	10.466
QALY (years)	7.621	7.057	7.411
Costs (RMB)	405,148	362,652	476,575
Incremental LY (years)		0.772	0.207
Incremental QALY (years)		0.564	0.211
Incremental total cost (RMB)		42,497	− 71,427
ICER (RMB/LY)		55,047	Dominant
ICUR (RMB/QALY)		75,349	Dominant

*CV* cardiovascular, *ICUR* incremental cost-utility ratio, *ICER* incremental cost-effectiveness ratio, *LY* Life-years, *QALY* quality-adjusted life-years, *SoC* standard of care

**Fig. 2:**
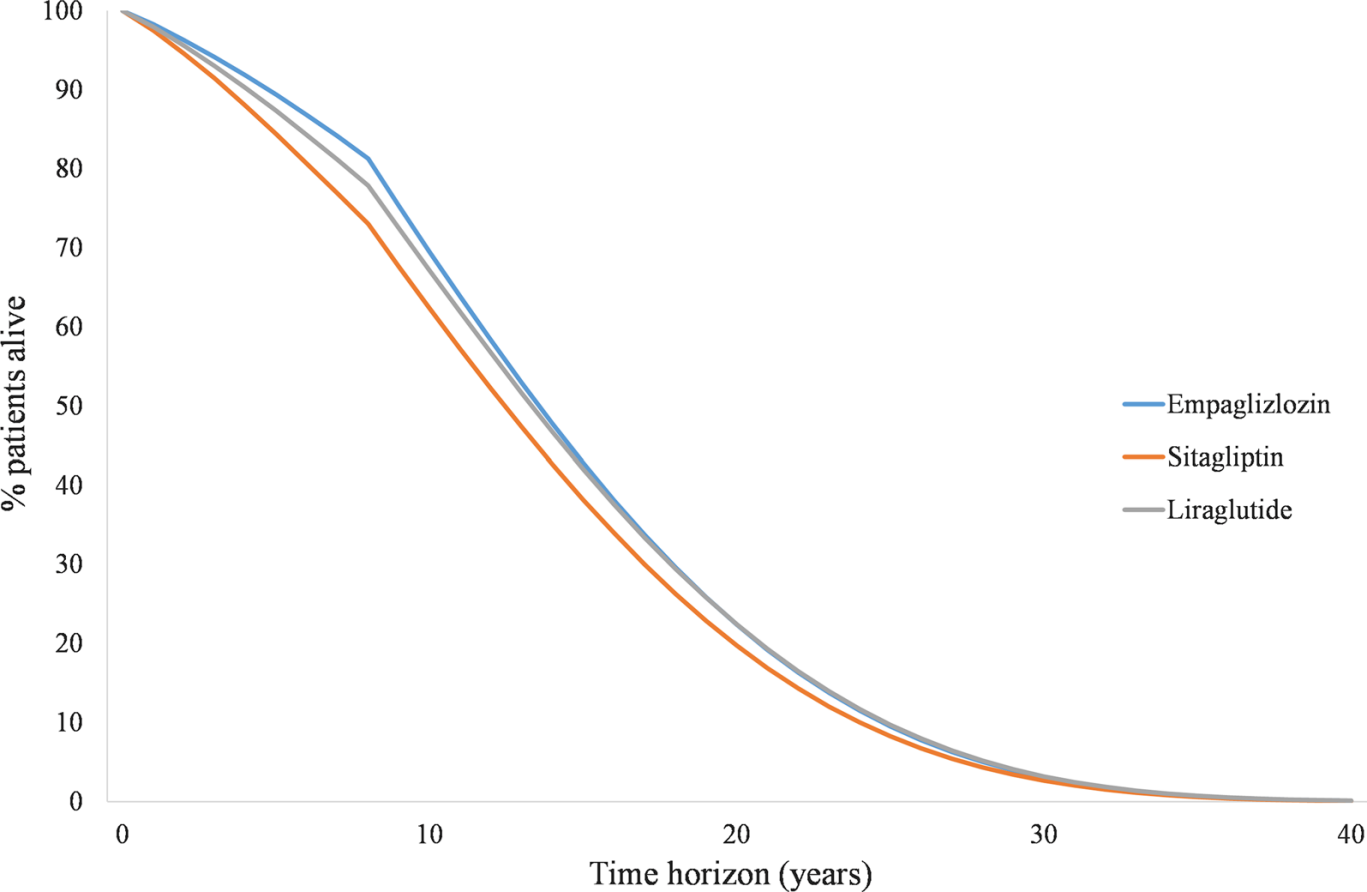
Lifetime survival of all therapies

In the initial years of the analysis, use of empagliflozin + SoC was associated with better survival as shown in Fig. 2.

In the long-term assessment, patients receiving empagliflozin + SoC would have a lower risk of HF compared to sitagliptin + SoC and liraglutide + SoC and have lower risk of gross renal proteinuria (GRP) and ESRD compared to sitagliptin + SoC.

As survival is better, patients receiving empagliflozin + SoC also bear higher costs in treating CVD, ulcers and eye disease complications compared to patients on either sitagliptin + SoC or liraglutide + SoC. Conversely, these patients present with lower costs in treating renal disease complications. Patients receiving liraglutide + SoC have the highest risk/cost of hypoglycemia.

### Scenario analysis

The cost-effectiveness analysis results for the five alternate scenarios are presented in Additional file 6. In the first four scenarios (excluding insulin costs in first line, reducing the time horizon to 5 years, apply the effects on cardiovascular outcomes only for 3 years, and having rescue therapy started at HbA1c of 9%) do not change the outcomes. Empagliflozin + SoC remains cost-effective versus sitagliptin + SoC and dominant versus liraglutide + SoC.

The CE results when not limiting the effects of liraglutide + SoC to eight years but having them applied until 8.5% HbA1c is reached, at 13 years. In this scenario (Scenario 5), empagliflozin + SoC is no longer increasing LYs and QALYs, while still resulting in cost-savings. Thus, results are located in the so-called South-Western quadrant of the cost-effectiveness plane. To be considered cost-effective in this quadrant the ICUR is to be higher than the threshold of 212,676 RMB, and this is the case with a value of 723,155 RMB. As such, empagliflozin + SoC is no longer dominant, but still cost-effective.

### Probabilistic sensitivity analysis

**Fig. 3:**
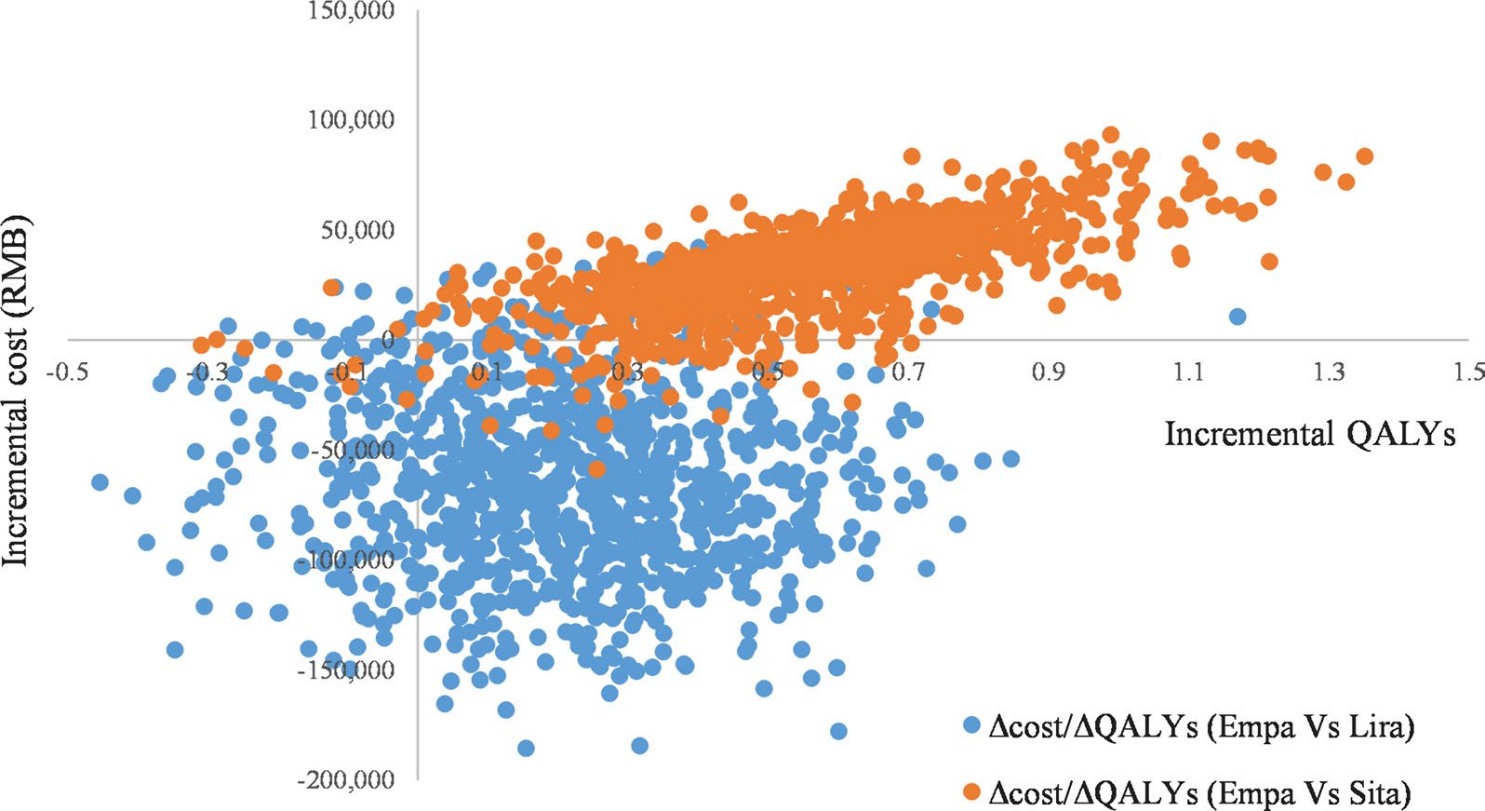
Cost-effectiveness scatter plot of empagliflozin + SoC versus sitagliptin + SoC and liraglutide + SoC (in terms of QALYs). *Empa* empagliflozin, *Lira* liraglutide, *QALE* quality-adjusted life expectancy, *QALY* quality-adjusted life-year, *Sita* sitagliptin, *SoC* standard of care

**Fig. 4:**
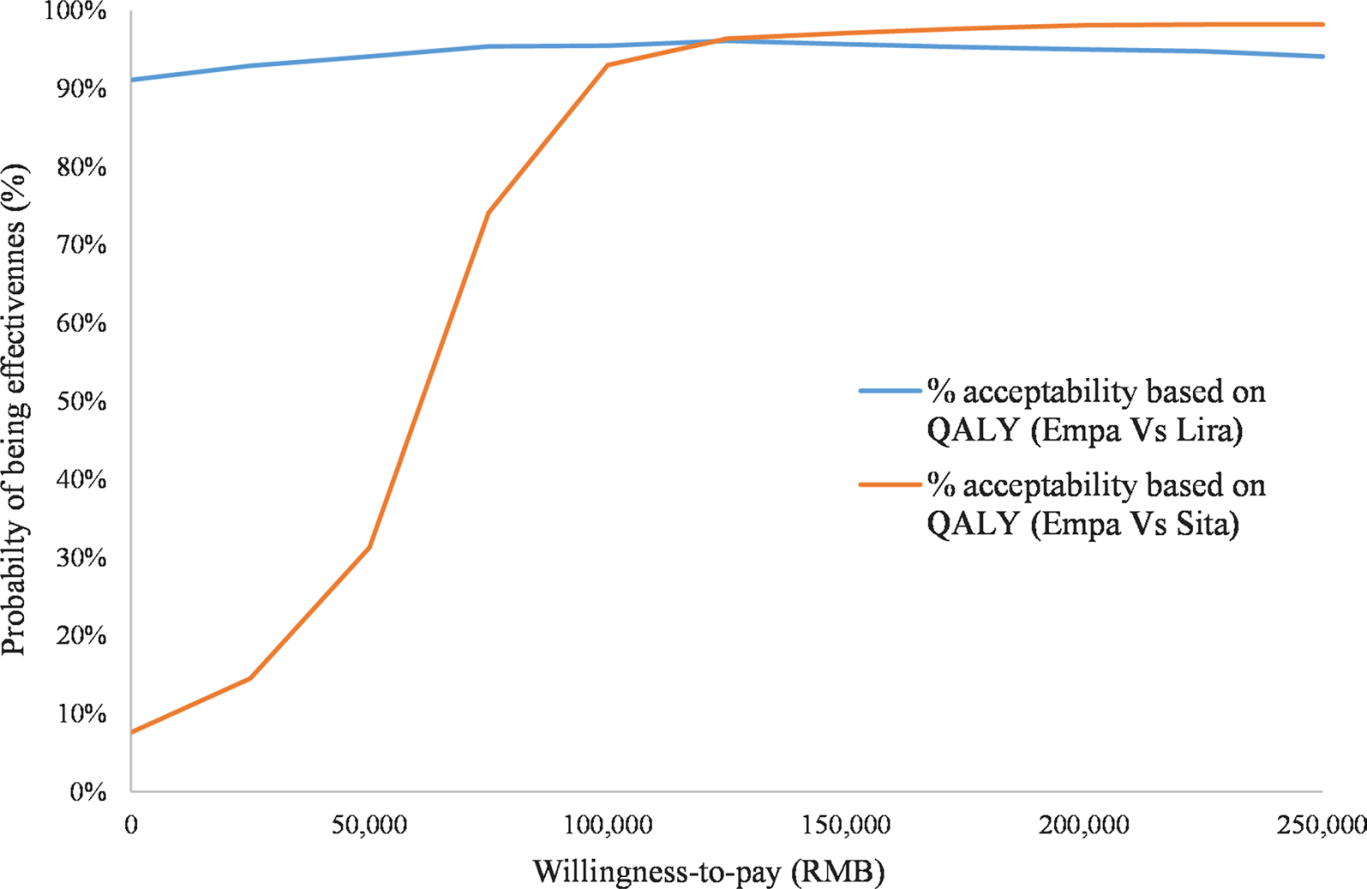
Cost-effectiveness acceptability curve of empagliflozin + SoC versus sitagliptin + SoC and liraglutide + SoC. *Empa* empagliflozin, *Lira* liraglutide, *QALE* quality-adjusted life expectancy, *Sita* sitagliptin, *SoC* standard of care

The PSA were performed on the base case to explore the impact of the combined uncertainty of all key parameters considered in the model on the ICUR. The main outputs of the PSA are the incremental CE scatter plots (Fig. 3) and the CE acceptability curves (Fig. 4).

Empagliflozin + SoC is cost-effective compared to sitagliptin + SoC in more than 98% of the simulations for a willingness-to-pay threshold of 212,676 RMB per QALY in Chinese health care perspective.

Empagliflozin + SoC is cost-effective versus liraglutide + SoC in 95% of the iterations considering a threshold of 212 K RMB per QALY from a Chinese health care perspective. Dominance of empagliflozin + SoC is observed in 76.4% of the iterations.

## Discussion

This is the second cost-effectiveness analysis regarding the use of empagliflozin + SoC in patients with T2D and established cardiovascular disease in China. A customized version of CDM was prepared to conduct this type of analysis and reflect the outcomes of different CVOTs.

For the current analysis, we opted for the UKPDS 82 cardiovascular risk equations to predict lifetime health outcomes after treatment switch because the calibration exercise showed that this option was predicting closest the EMPA-REG OUTCOME trial outcomes, and also considers most of the surrogate endpoints described in the study.

The current analysis on CE of empagliflozin + SoC based on CVOT outcomes showed that empagliflozin + SoC provides additional LY (+ 0.772) and QALY (+ 0.564) for an incremental cost of 42,497 RMB (US$6,053) compared to sitagliptin + SoC. As a result, the ICER of empagliflozin + SoC compared to sitagliptin + SoC is 55,047 RMB; and the ICUR compared to sitagliptin + SoC is 75,349 RMB (US$10,732) as per Chinese healthcare perspective. Both ICER and ICUR are below the willingness-to-pay threshold of 212,676 RMB, corresponding to the Gross Domestic Product in China in 2018 and seen by the WHO as a possible threshold to consider the intervention cost-effective. Sitagliptin + SoC has a lower HbA1c decline in the first year compared to empagliflozin + SoC and liraglutide + SoC, and thus the HbA1c in sitagliptin + SoC patients progresses faster compared to liraglutide + SoC and empagliflozin + SoC. This finding can explain the lower LYs and QALYs observed in sitagliptin + SoC treatment arm.

Compared to liraglutide + SoC, empagliflozin + SoC use leads to additional 0.207 LY and 0.211 QALY gains and results in cost-savings of 71,427 RMB (US$10,173). Thus, in this comparison, empagliflozin + SoC can be considered a dominant alternative.

In the short-term analysis (time horizon of 5 years), the clinical benefits of empagliflozin + SoC were demonstrated in avoiding death due to CV and non-CV causes, HF, MI, eye disease complications, GPR and ESRD. Whereas in the long-term analyses, these benefits are partly offset by the lower mortality in the empagliflozin arm which results in a longer time that diabetes complications can happen, and costs can occur (“survival paradox”). As a result, complication, treatment, and management costs increased in the empagliflozin + SoC arm.

It is worth noting a few key assumptions that were incorporated into the model. Firstly, an HbA1c threshold of 8.5% was applied despite the fact that treatment guidelines recommend HbA1c thresholds of 7% or 7.5% for treatment switching. The reason for choosing this threshold was that, the baseline HbA1c value was 8.1% and the treatment effect with empaglilozin + SoC was − 0.58% during the first year. Therefore, testing a lower threshold would not be appropriate, as patients would not reach the official threshold. Also, during the different CVOTs, concomitant treatments were adapted and doses increased and as such considered in the model’s HbA1c progression and costing until patients switched to rescue treatment.

Secondly, patients were receiving a broad mix of therapies in CVOT, and continuous adaptations to treatment were required to keep HbA1c low according to local guidelines. To address this, a high dose of basal bolus insulin was chosen as the next line therapy when the combined therapies were administered to patients in the CVOT. It should also be noted that renal outcomes were not included in the ITCs, whereas a clear treatment effect on renal function was observed in the EMPA-REG OUTCOME study. Given that the sitagliptin + SoC CVOT did not assess the treatment benefit on the renal function, the observed outcomes on microalbuminuria and ESRD for sita was assumed to be equal to the placebo arm in the EMPA-REG OUTCOME study. In the LEADER study, liraglutide + SoC showed an impact on renal disease and in this cost-effectiveness analysis, we assumed that impact was the same as for empagliflozin + SoC.

Scenario analyses evaluated the effects of treatments at a shorter time horizon (i.e. 5 years), applying CV outcomes only for 3 years (i.e. the trial follow-up period), applying treatment intensification at a HbA1c threshold of 9%; and when excluding insulin costs. The results confirmed the cost-effective profile of empagliflozin + SoC against sitagliptin + SoC and its dominant profile against liraglutide + SoC. Only when the effect of liraglutide + SoC was prolonged to 13 years, empagliflozin + SoC is no longer dominant, but still cost-effective versus liraglutide + SoC.

The results of the PSA showed that in most of the simulations (at least 90%), empagliflozin + SoC would be a cost-effective therapy for a willingness-to-pay inferior to 212,676 RMB.

Only one study evaluating the cost-effectiveness of empagliflozin versus standard of care in adult patients with T2D and high risk of CVD in Chinese population was identified in the public domain [18]. This study was conducted using a patient level simulation model developed in MS Excel, based on the EMPA-REG OUTCOME trial data, and assessed rates of outcome, events, costs, LYs, QALYs, and ICER. In the study, treatment with empagliflozin on top of SoC resulted in an additional 1.01 QALYs versus SoC alone (7.04 QALYs) at an incremental cost of ¥ 4002 per patient. The ICUR was estimated at ¥ 3988 per QALY gained.

Apart from this, Zang et al. [35] and Mingxing et al. [36] reported clinical and safety data comparing liraglutide and sitagliptin as an add-on to metformin in Chinese patients with T2D without established CVD. Roden et al. [37] studied the efficacy and tolerability of empagliflozin versus sitagliptin in patients with T2D (Asian patients represented 64% of the study cohort) without any medication for the 12 preceding weeks. All 3 studies reported short outcomes for 24–26 weeks of treatment. However, none of these studies were head-to-head analysis comparing empagliflozin, liraglutide, and sitagliptin in patients with established CVD. Therefore, the ITC was the only available source to perform this analysis, which considered data EMPA-REG OUTCOME trial and two CVOTs on sitagliptin + SoC and liraglutide + SoC [22, 23].

Clinical data used in the modeling study were generated via a multi-country study and are non-specific for the Chinese population with T2D with established CVD. It is generally known that CVD manifests differently in Asian population in comparison to Western population (stroke versus MI ratio). Nevertheless, the EMPA-REG OUTCOME [37] and TECOS trials [38] included more than 20% of Asian individuals each and in LEADER [39] almost 10% of patients were Asians. Although we recognize this as a limitation of the study, many other health economic (HE) studies published Chinese findings using clinical data of trial populations not involving Asians or Chinese patients [40, 41].

There is a limited choice of next-line therapy in China after a combination of empagliflozin, or liraglutide, or sitagliptin with background therapies that already included other oral antidiabetic drugs and insulin, therefore, we opted for a combination of basal and bolus insulin as reported by Riddle et al. [24]. In this study glargine was used as the basal insulin, whereas in Asia and in China in particular, NPH is the commonly used insulin, which is much cheaper than glargine. Nevertheless, this would not have an impact on study outcomes as empagliflozin was already cost-effective at 5 years, before the treatment intensification.

Lastly, the present study was run using a calibrated version of the CDM, developed to predict the outcomes of the different CVO trials and of the ITC in the analyses. This process included a validation step, to ensure that the methodology used would enable CDM to predict the trials' outcomes accurately at 3 years.

## Conclusion

This study demonstrated that empagliflozin + SoC is a cost-effective treatment compared with sitagliptin + SoC, while being a dominant therapy against liraglutide + SoC in the management of T2DM patients with established CVD. Sensitivity analyses confirmed the robustness of these findings.

## Supplementary Information

**Additional file 1. **Assumptions applied to match endpoint definitions in ITC and CDM.

**Additional file 2. **Indirect comparison [RR (95% CI)] of empagliflozin + SoC versus other glucose-lowering drugs. To match the endpoints specified in the EMPA REG OUTCOME trial and those reported by the ITCs with the CDM endpoints, some assumptions were made, which are summarized in Additional file 1 and this file.

**Additional file 3. **Baseline characteristics of the EMPA-REG OUTCOME study. It shows the cohort under analysis represents the characteristics of patient with T2D and established CVD at baseline, and as such, the baseline characteristics of patients in the EMPA-REG OUTCOME trial were used.

**Additional file 4. **Chinese diabetes complication related costs (2019, RMB). It presents direct medical costs included pharmacy costs, management cost (screening test, concomitant medication) and the costs of T2D complications.

**Additional file 5. **Default utilities/disutilities in the IQVIA Core Diabetes Model. Starting from a Chinese specific baseline utility, the default CDM utility data associated with events and health states were applied which is presented in this file.

**Additional file 6. **Cost-effectiveness results for the scenario analyses. The cost-effectiveness analysis results for the five alternate scenarios are presented in this file.

## Data Availability

Not applicable.

## Abbreviations

ACE: Angiotensin-converting enzyme
ACM: All-cause mortality
ARB: Angiotensin receptor blocker
BMI: Body mass index
CDM: Core Diabetes Model
CE: Cost-effectiveness
CEAC: Cost-effectiveness acceptability curves
CVD: Cardiovascular disease
CVOT: Cardiovascular outcome trials
DPP4 inhibitors: Dipeptidyl peptidase 4 inhibitors
EMA: European Medicines Agency
EMPA: Empagliflozin
ESRD: End-stage renal disease
FDA: Food and Drug Administration
F&NF: Fatal and non-fatal
GLP-1: Receptor agonists glucagon-like peptide 1 receptor agonists
GRP: Gross renal proteinuria
HF: Heart failure
ICER: Incremental cost-effectiveness ratio
ICUR: Incremental cost-utility ratios
ITC: Indirect treatment comparisons
KOL: Key opinion leader
LYs: Life-years
MAU: Microalbuminuria
MI: Myocardial infarction
NSHE: Non-severe hypoglycemic event
OAD: Oral anti-diabetics
PSA: Probabilistic sensitivity analysis
QALYs: Quality adjusted life years
SHE: Severe hypoglycemic event
SoC: Standard of care
T2D: Type 2 diabetes
UKPDS: United Kingdom Prospective Diabetes Study
US: United States
WHO: World Health Organization
