# Supplementary material for: Cost-effectiveness of empagliflozin in patients with type 2 diabetes and established cardiovascular disease in China
Source: Cost Eff Resour Alloc. 2021 Aug 4;19:46. doi: 10.1186/s12962-021-00299-z (PMC8336098; doi:10.1186/s12962-021-00299-z)
Supplement: Supplementary file 1 — Additional file 1. Assumptions applied to match endpoint definitions in ITC and CDM. [file 12962_2021_299_MOESM1_ESM.docx]

**Table S1. Assumptions applied to match endpoint definitions in ITC and CDM**

| **ITC endpoint** | **CDM endpoint** | **Comment** |
| --- | --- | --- |
| CV-related mortality | Event fatality | The UKPDS 82 mortality equations are not specifically designed to capture CV mortality. Hence, when UKPDS 82 mortality equations are applied, the CDM predicts the incidence of event fatality and non-event related fatality. As a proxy, we compared CV mortality endpoint from the ITC to event fatality, i.e. mortality in the year of one of the following events: MI, Stroke, HF, angina, ESRD or amputation. |
| ACM | Mortality from all causes | Mortality from all causes was assessed in the CDM as the sum of event fatality and non-event related fatality. Since event fatality risk was already adjusted by the RR from the above category, the RR for the non-event related fatality risk (i.e. fatality related to the history of complications in subsequent years or non-diabetes specific fatality) was calibrated such that the ACM outcome from the CDM matched the expected ACM outcome for the respective comparator following ITC adjustments. |
| Composite endpoint | */* | The composite endpoint is not evaluated in the CDM |
| Hospitalization due to HF | HF | Assuming hospitalization due to heart failure was equal to the HF definition in the CDM. |
| NF-stroke | F&NF stroke | Since the CDM predicts the incidence of fatal & non-fatal stroke, the RR for NF-stroke from the ITC was adjusted to derive the corresponding RR for F&NF stroke according to the proportions of NF-stroke and F stroke in the EMPA arm of the EMPA-REG OUTCOME study. This adjustment was based on the assumption that the RR for F stroke was risk neutral (i.e. RR = 1). As CV mortality is already considered in the first endpoint of this list, this assumption was applied to avoid double counting.  Example calculation for LIRA:   - % NF-stroke in EMPA = 91.5% - % F stroke in EMPA = 8.5% - ITC predicted RR NF-stroke (EMPA vs. LIRA) = 1.39 - Adjusted RR F&NF-stroke (EMPA vs. LIRA) = 1.39 * 91.5% + 1.0 * 8.5% = 1.36 |
| NF MI | F&NF MI | The same approach as presented in the above example for stroke was applied to MI following 95.5% NF MI events and 4.5% F MI events in the EMPA arm of the EMPA-REG OUTCOME study. |

ACM=all-cause mortality; CDM=Core Diabetes Model; CV=cardiovascular; EMPA=empagliflozin; ESRD= end-stage renal disease; F=fatal; F&NF= NF=fatal and non‑fatal; GRP=gross proteinuria, HF=heart failure, ITC=indirect treatment comparison; LIRA=liraglutide; MAU=microalbuminuria, MI=myocardial infarction; NF=non‑fatal; RR=relative risk; SoC=standard of care; UKPDS=United Kingdom Prospective Diabetes Study
