# Supplementary material for: Cost-effectiveness of empagliflozin in patients with type 2 diabetes and established cardiovascular disease in China
Source: Cost Eff Resour Alloc. 2021 Aug 4;19:46. doi: 10.1186/s12962-021-00299-z (PMC8336098; doi:10.1186/s12962-021-00299-z)
Supplement: Supplementary file 2 — Additional file 2. Indirect comparison [RR (95% CI)] of empagliflozin + SoC versus other glucose-lowering drugs. To match the endpoints specified in the EMPA REG OUTCOME trial and those reported by the ITCs with the CDM endpoints, some assumptions were made, which are summarized in Additional file 1 and this file. [file 12962_2021_299_MOESM2_ESM.docx]

**Table S2. Indirect comparison [RR (95% CI)] of empagliflozin+SoC versus other glucose-lowering drugs**

| **Comparison of Empagliflozin+SoC *vs*** | **Cardiovascular- related mortality** | **All-cause mortality** | **Composite endpoint** | **Hospitalization due to heart failure** | **Non-fatal stroke** | **Non-fatal myocardial infarction** |
| --- | --- | --- | --- | --- | --- | --- |
| Sitagliptin+SoC | 0.60 (0.46, 0.79) | 0.67 (0.54, 0.83) | 0.87 (0.73, 1.04) | 0.65 (0.47, 0.90) | -- | -- |
| Liraglutide+SoC | 0.80 (0.60, 1.06) | 0.80 (0.64, 1.00) | 0.99 (0.82, 1.18) | 0.75 (0.54, 1.03) | 1.39 (0.97, 2.01) | 0.99 (0.76, 1.30) |

CI: confidence interval; RR: relative risk; SoC=standard of care
