# Supplementary material for: Cost-effectiveness of empagliflozin in patients with type 2 diabetes and established cardiovascular disease in China
Source: Cost Eff Resour Alloc. 2021 Aug 4;19:46. doi: 10.1186/s12962-021-00299-z (PMC8336098; doi:10.1186/s12962-021-00299-z)
Supplement: Supplementary file 3 — Additional file 3. Baseline characteristics of the EMPA-REG OUTCOME study. It shows the cohort under analysis represents the characteristics of patient with T2D and established CVD at baseline, and as such, the baseline characteristics of patients in the EMPA-REG OUTCOME trial were used. [file 12962_2021_299_MOESM3_ESM.docx]

**Table S3. Baseline characteristics of the EMPA-REG OUTCOME study**

|  | **Value** | | **Units/Range** |
| --- | --- | --- | --- |
| **PATIENT DEMOGRAPHICS** | Mean | SE/SD |  |
| Start age | 63.10 | 8.60 | years |
| Duration of Diabetes | 9 | 0.00 | years |
| Prop. Male | 0.71 |  | [0-1] |
| **BASELINE RISK FACTORS** | Mean | SE/SD |  |
| HbA1c | 8.07 | 0.85 | %-points |
| SBP | 135.47 | 17.00 | mmHg |
| DBP | 76.67 | 9.83 | mmHg |
| T-CHOL | 162.90 | 43.80 | mg/dL |
| HDL | 44.40 | 11.70 | mg/dL |
| LDL | 85.60 | 35.70 | mg/dL |
| TRIG | 170.50 | 126.90 | mg/dL |
| BMI | 30.62 | 5.26 | kg/m^2^ |
| eGFR | 74.0aa4 | 21.41 | ml/min/ 1.73m^2^ |
| HAEM | 14.5 | 0 | gr/dl |
| WBC | 6.8 | 0 | 106/ml |
| Heart rate | 68.47 | 0 | bpm |
| WHR | 0.93 | 0 | (1 unit) |
| uAER | 19.3 | 2.9 | mg/mmol |
| Serum creatinine | 1.1 | 0 | mg/dl |
| Serum albumin | 3.9 | 0 | g/dl |
| Prop. smoker | 0.132 |  | [0-1] |
| Cigarettes/day | 3 |  |  |
| Alcohol consumption | 3 |  | Oz/week |
| **RACIAL CHARACTERISTICS** | Mean |  |  |
| Prop. White | 0 |  | [0-1] |
| Prop. Black | 0 |  | [0-1] |
| Prop. Hispanic | 0 |  | [0-1] |
| Prop. Native American | 0 |  | [0-1] |
| Prop. Asian/Pacific Islander | 1 |  | [0-1] |
| **BASELINE CVD COMPLICATIONS** | Mean |  |  |
| Prop. Myocardial infarction | 0.466 |  | [0-1] |
| Prop. Angina | 0.290 |  | [0-1] |
| Prop. Peripheral Vascular Disease | 0.208 |  | [0-1] |
| Prop. Stroke | 0.233 |  | [0-1] |
| Prop. Heart failure | 0.101 |  | [0-1] |
| **BASELINE RENAL COMPLICATIONS** | Mean |  |  |
| Prop. Macular edema | 0.195 |  | [0-1] |
| **BASELINE RETINOPATHY COMPLICATIONS** | Mean |  |  |
| Prop. Background diabetic retinopathy | 0.110 |  | [0-1] |
| Prop. Proliferative diabetic retinopathy | 0.110 |  | [0-1] |
| **BASELINE FOOT ULCER COMPLICATIONS** | Mean |  |  |
| Prop. uninfected ulcer | 0.006 |  | [0-1] |
| **BASELINE NEUROPATHY** | Mean |  |  |
| Prop. neuropathy | 0.313 |  | [0-1] |

SE=standard error; SD=standard deviation; HbA1c=hemoglobin A1c; SBP=systolic blood pressure; DBP=diastolic blood pressure; T-Chol=total cholesterol; HDL=high-density lipoprotein; LDL=low-density lipoprotein; TRIG=triglycerides; BMI=body mass index; eGFR=estimated glomerular filtration rate; HAEM=hemoglobin; WBC=white blood cell count; WHR=waist‑to‑hip ratio; uAER=urinary albumin creatinine ratio; ser_creat=serum creatinine; Ser_alb=serum albumin
