# Supplementary material for: Cost-effectiveness of empagliflozin in patients with type 2 diabetes and established cardiovascular disease in China
Source: Cost Eff Resour Alloc. 2021 Aug 4;19:46. doi: 10.1186/s12962-021-00299-z (PMC8336098; doi:10.1186/s12962-021-00299-z)
Supplement: Supplementary file 4 — Additional file 4. Chinese diabetes complication related costs (2019, RMB). It presents direct medical costs included pharmacy costs, management cost (screening test, concomitant medication) and the costs of T2D complications. [file 12962_2021_299_MOESM4_ESM.docx]

**Table S4. Chinese diabetes complication related costs (2019, RMB)**

| **Variable** | **Cost (RMB)** | **Source** |
| --- | --- | --- |
| **Management costs** | | |
| Annual statins | 1068.93 | Chinese Simvastatin daily costs (local hospital) ×365 |
| Annual aspirin | 219.00 | Chinese aspirin daily costs (local hospital) ×365 |
| Annual ACE inhibitors/ARBs | 47.45 | Chinese Ramipril daily costs (local hospital) ×365 |
| Annual screening microalbuminuria | 40.00 | Local KOL survey |
| Annual screening gross proteinuria | 15.00 | Local KOL survey |
| Stopping ACEs due to side effects | 0.00 | Assumption that no additional costs are incurred |
| Annual eye screening | 60.00 | Local KOL survey |
| Annual foot screening program | 0.00 | Assumed to be routine part of care (set to 0) |
| **Complication Costs** | | |
| CVD complications* |  |  |
| Myocardial infarction 1^st^ year | 31,835.00 | [[26](#_ENREF_26)] |
| Myocardial infarction 2+ years | 10,612.00 | [[26](#_ENREF_26)] |
| Angina 1^st^ year | 40,380.00 | [[26](#_ENREF_26)] |
| Angina 2+ years | 7,209.00 | [[26](#_ENREF_26)] |
| Congestive heart failure 1^st^ year | 9,551.00 | [[26](#_ENREF_26)] |
| Congestive heart failure 2+ years | 6,367.00 | [[26](#_ENREF_26)] |
| Stroke 1^st^ year | 18,763.00 | [[26](#_ENREF_26)] |
| Stroke 2+ years | 8,449.00 | [[26](#_ENREF_26)] |
| Stroke death | 14,541.00 | [[26](#_ENREF_26)] |
| Peripheral vascular disease 1^st^ year | 24,858.00 | [[26](#_ENREF_26)] |
| Peripheral vascular disease 2+ years | 9,662.00 | [[26](#_ENREF_26)] |
| Renal Complications* |  |  |
| Hemodialysis costs 1^st^ year | 83,497.00 | [[26](#_ENREF_26)] |
| Hemodialysis costs 2+ years | 70,491.00 | [[26](#_ENREF_26)] |
| Peritoneal dialysis costs 1^st^ year | 57,927.00 | [[26](#_ENREF_26)] |
| Peritoneal dialysis costs 2+ years | 47,247.00 | [[26](#_ENREF_26)] |
| Renal transplant costs 1^st^ year | 250,832.00 | [[26](#_ENREF_26)] |
| Renal transplant costs 2+ years | 66,334.00 | [[26](#_ENREF_26)] |
| Adverse events |  |  |
| Severe hypoglycemia needing medical assistance (SHE 2) | 3,788.00 | [[26](#_ENREF_26)] |
| Severe hypoglycemia not needing medical assistance (SHE 1) | 178.00 | [[26](#_ENREF_26)] |
| NSHE | 178.00 | [[26](#_ENREF_26)] |
| Urinary tract infection + genital infection | 9.00 | Chinese Procurement Website |
| Eye disease |  |  |
| Laser treatment | 2,341.00 | [[26](#_ENREF_26)] |
| Cataract operation | 7,371.00 | [[26](#_ENREF_26)] |
| Following cataract operation | 193.00 | [[26](#_ENREF_26)] |
| Blindness – year of onset | 5,520.00 | [[26](#_ENREF_26)] |
| Blindness – following years | 1,496.00 | [[26](#_ENREF_26)] |
| Neuropathy |  |  |
| Neuropathy 1^st^ year | 5,234.00 | [[26](#_ENREF_26)] |
| Neuropathy 2+ years | 6,343.00 | [[26](#_ENREF_26)] |
| Amputation (event based) | 6,979.00 | [[26](#_ENREF_26)] |
| Amputation prosthesis (event based) | 21,010.00 | [[26](#_ENREF_26)] |
| Gangrene treatment | 4,653.00 | [[26](#_ENREF_26)] |
| After healed ulcer | 877.00 | Local KOL survey |
| Infected ulcer | 5,234.00 | [[26](#_ENREF_26)] |
| Standard uninfected ulcer | 3,194.00 | [[26](#_ENREF_26)] |
| Healed ulcer history of amputation | 877.00 | Local KOL survey |

ACE=angiotensin-converting enzyme; ARB=angiotensin receptor blocker; CVD=cardiovascular disease; KOL=key opinion leader; NSHE=non-severe hypoglycemic event; SHE=severe hypoglycemic event SoC=standard of care
