# Supplementary material for: Cost-effectiveness of empagliflozin in patients with type 2 diabetes and established cardiovascular disease in China
Source: Cost Eff Resour Alloc. 2021 Aug 4;19:46. doi: 10.1186/s12962-021-00299-z (PMC8336098; doi:10.1186/s12962-021-00299-z)
Supplement: Supplementary file 5 — Additional file 5. Default utilities/disutilities in the IQVIA Core Diabetes Model. Starting from a Chinese specific baseline utility, the default CDM utility data associated with events and health states were applied which is presented in this file. [file 12962_2021_299_MOESM5_ESM.docx]

**Table S5: Default utilities/disutilities in the Core Diabetes Model**

| **Variables** | **Value** | **Source** |
| --- | --- | --- |
| U T2 no complications | 0.876 | [[27](#_ENREF_27)] |
| **DisU MI event** | **-0.055** | [[28](#_ENREF_28)] |
| U post MI | 0.730 | [[28](#_ENREF_28)] |
| U angina | 0.695 | [[28](#_ENREF_28)] |
| U CHF | 0.677 | [[28](#_ENREF_28)] |
| **DisU stroke event** | **-0.164** | [[28](#_ENREF_28)] |
| U post Stroke | 0.621 | [[28](#_ENREF_28)] |
| U PVD | 0.724 | [[28](#_ENREF_28)] |
| U MA | 0.785 | [[28](#_ENREF_28)] |
| U GRP | 0.737 | [[28](#_ENREF_28)] |
| U HD | 0.621 | [[28](#_ENREF_28)] |
| U PD | 0.581 | [[28](#_ENREF_28)] |
| U RT | 0.762 | [[28](#_ENREF_28)] |
| U BDR | 0.745 | [[28](#_ENREF_28)] |
| U PDR laser treated | 0.715 | [[28](#_ENREF_28)] |
| U PDR no Laser | 0.715 | [[28](#_ENREF_28)] |
| U ME | 0.745 | [[28](#_ENREF_28)] |
| U SVL | 0.711 | [[28](#_ENREF_28)] |
| U cataract | 0.769 | [[28](#_ENREF_28)] |
| U neuropathy | 0.701 | [[28](#_ENREF_28)] |
| U healed ulcer | 0.785 | [[28](#_ENREF_28)] |
| U active ulcer | 0.615 | [[28](#_ENREF_28)] |
| U post amputation | 0.505 | [[28](#_ENREF_28)] |
| **DisU GUI** | **0** |  |
| U post GUI | 0.785 |  |
| Diminishing NSHE disutility | yes | [[35](#_ENREF_35)] |
| DisU for SHE 1 (during daytime) | -0.014 | [[26](#_ENREF_26)] |
| DisU for SHE 1 (nocturnal) | -0.014 | [[26](#_ENREF_26)] |
| DisU for SHE 2 (during daytime) | -0.058 | [[26](#_ENREF_26)] |
| DisU for SHE 2 (nocturnal) | -0.058 | [[26](#_ENREF_26)] |

* = No state-specific health utility identified – conservatively assumed to be equivalent to complication-free utility; 0.020 = disutility for mild vision loss; 0.190 = disutility for neuropathy
