# Supplementary material for: Cost-effectiveness of empagliflozin in patients with type 2 diabetes and established cardiovascular disease in China
Source: Cost Eff Resour Alloc. 2021 Aug 4;19:46. doi: 10.1186/s12962-021-00299-z (PMC8336098; doi:10.1186/s12962-021-00299-z)
Supplement: Supplementary file 6 — Additional file 6. Cost-effectiveness results for the scenario analyses. The cost-effectiveness analysis results for the five alternate scenarios are presented in this file. [file 12962_2021_299_MOESM6_ESM.docx]

**Table S6. Cost-effectiveness results for the scenario analyses**

| **Parameter** | **Empagliflozin+SoC** | **Sitagliptin+SoC** | **Liraglutide+SoC** |
| --- | --- | --- | --- |
| ***Scenario 1: Excluding insulin costs*** | | | |
| LY (years) | 10.673 | 9.901 | 10.466 |
| QALY (years) | 7.621 | 7.057 | 7.411 |
| Incremental LY (years) |  | 0.772 | 0.207 |
| Incremental QALY (years) |  | 0.564 | 0.211 |
| Incremental total cost (RMB) |  | 22,992 | -54,440 |
| ICER (RMB/LY) |  | 29,782 | Dominant |
| ICUR (RMB/QALY) |  | 40,765 | Dominant |
| ***Scenario 2:*** ***Time horizon of 5 years*** | | | |
| LY (years) | 4.308 | 4.188 | 4.258 |
| QALY (years) | 3.211 | 3.11 | 3.108 |
| Incremental LY (years) |  | 0.120 | 0.051 |
| Incremental QALY (years) |  | 0.100 | 0.102 |
| Incremental total cost (RMB) |  | 12,736 | -57,626 |
| ICER (RMB/LY) |  | 106,136 | Dominant |
| ICUR (RMB/QALY) |  | 127,363 | Dominant |
| ***Scenario 3:*** ***CV outcomes applied for 3 years only*** | | | |
| LY | 9.984 | 9.647 | 10.072 |
| QALY | 7.161 | 6.896 | 7.149 |
| Incremental LY |  | 0.337 | -0.088 |
| Incremental QALY |  | 0.264 | 0.012 |
| Incremental total cost (RMB) |  | 24,134 | -82,738 |
| ICER (RMB/LY) |  | 71,615 | 940,206 |
| ICUR (RMB/QALY) |  | 91,418 | Dominant |
| ***Scenario 4: HbA1c threshold to switch therapy at 9%*** | | | |
| LY | 11.2 | 10.055 | 10.823 |
| QALY | 8.064 | 7.22 | 7.701 |
| Incremental LY |  | 1.145 | 0.377 |
| Incremental QALY |  | 0.844 | 0.362 |
| Incremental total cost (RMB) |  | 55,908 | -94,044 |
| ICER (RMB/LY) |  | 48,828 | Dominant |
| ICUR (RMB/QALY) |  | 66,242 | Dominant |
| ***Scenario 5: extending liraglutide+SoC CVO effects up to 13 years*** | | | |
| LY | 10.673 |  | 10.901 |
| QALY | 7.621 |  | 7.764 |
| Incremental LY |  |  | -0.228 |
| Incremental QALY |  |  | -0.142 |
| Incremental total cost (RMB) |  |  | -94,044 |
| ICER (RMB/LY) |  |  | 450,361 |
| ICUR (RMB/QALY) |  |  | 723,115 |

LY=life-year; QALY=quality-adjusted life-year; ICUR=incremental cost-utility ratio; ICER=incremental cost-effectiveness ratio
